# Myrtle-Functionalized Nanofibers Modulate Vaginal Cell Population Behavior While Counteracting Microbial Proliferation

**DOI:** 10.3390/plants11121577

**Published:** 2022-06-15

**Authors:** Emanuela Bellu, Nicia Diaz, Martin Kralovič, Radek Divin, Giorgia Sarais, Angela Fadda, Rosanna Satta, Maria Antonia Montesu, Serenella Medici, Antonio Brunetti, Ana Rita Pinheiro Barcessat, Taťána Jarošíková, Jiří Rulc, Evzen Amler, Valentina Margarita, Paola Rappelli, Margherita Maioli

**Affiliations:** 1 Department of Biomedical Sciences, University of Sassari, Viale San Pietro 43/B, 07100 Sassari, Italy; ema.bellu@hotmail.it (E.B.); ndiaz@uniss.it (N.D.); brunetti@uniss.it (A.B.); ritabarcesat@gmail.com (A.R.P.B.); vmargarita@uniss.it (V.M.); rappelli@uniss.it (P.R.); 2 Institute of Biophysics, 2nd Faculty of Medicine, Charles University, V Uvalu 84, 15006 Prague, Czech Republic; mkralovic@centrum.cz (M.K.); radek.divin@cvut.cz (R.D.); evzen.amler@lfmotol.cuni.cz (E.A.); 3 Department of Life and Environmental Sciences, University of Cagliari, Via Ospedale 72, 09124 Cagliari, Italy; gsarais@unica.it; 4 Istituto di Scienze delle Produzioni Alimentari (ISPA), Consiglio Nazionale delle Ricerche (CNR), Traversa la Crucca 3, 07100 Sassari, Italy; angela.fadda@cnr.it; 5 Department of Medical, Surgical and Experimental Sciences, University of Sassari, 07100 Sassari, Italy; rsatta@uniss.it (R.S.); mmontesu@uniss.it (M.A.M.); 6 Department of Chemistry and Pharmacy, University of Sassari, 07100 Sassari, Italy; sere@uniss.it; 7 Health and Biological Sciences Department, Federal University of Amapà, Macapa 68902280, Brazil; 8 Faculty of Biomedical Engineering, Czech Technical University in Prague, 27201 Prague, Czech Republic; jarostat@fbmi.cvut.cz; 9 Ambis College, Lindnerova 575/1, 18000 Prague, Czech Republic; jirka.rulc@centrum.cz; 10 UCEEB, Czech Technical University, Trinecka 1024, 27343 Bustehrad, Czech Republic; 11 Center for Developmental Biology and Reprogramming (CEDEBIOR), Department of Biomedical Sciences, University of Sassari, Viale San Pietro 43/B, 07100 Sassari, Italy

**Keywords:** myrtle, plant extracts, bioactive compounds, health promoting, nanomaterials, antimicrobial activity, cell behavior, vaginal infections

## Abstract

Vaginal infections affect millions of women annually worldwide. Therapeutic options are limited, moreover drug-resistance increases the need to find novel antimicrobials for health promotion. Recently phytochemicals were re-discovered for medical treatment. Myrtle (*Myrtus communis* L.) plant extracts showed in vitro antioxidant, antiseptic and anti-inflammatory properties thanks to their bioactive compounds. The aim of the present study was to create novel nanodevices to deliver three natural extracts from leaves, seeds and fruit of myrtle, in vaginal milieu. We explored their effect on human cells (*HeLa*, Human Foreskin Fibroblast-1 line, and stem cells isolated from skin), resident microflora (*Lactobacillus acidophilus*) and on several vaginal pathogens (*Trichomonas vaginalis*, *Escherichia coli*, *Staphylococcus aureus*, *Candida albicans*, *Candida kefyr*, *Candida glabrata*, *Candida parapsilosis*, *Candida krusei*). Polycaprolactone-Gelatin nanofibers encapsulated with leaves extract and soaked with seed extracts exhibited a different capability in regard to counteracting microbial proliferation. Moreover, these nanodevices do not affect human cells and resident microflora viability. Results reveal that some of the tested nanofibers are interesting candidates for future vaginal infection treatments.

## 1. Introduction

The vagina is an organ of female reproductive tract represented by a collapsed tube located in a woman’s pelvis, which extends from the external vaginal orifice to the cervix. The vaginal environment is comprised of different areas populated by various cell types. The vaginal mucosa is a squamous epithelium organized in different levels where cells become more differentiated, migrating apically [1]. Here, resident fibroblasts are responsible for the production of hyaluronic acid, thus maintaining hydration of the area [2].

The integrity of vaginal epithelium is essential for women’s reproductive health. It provides a barrier against HIV and sexually transmitted infections [3].

In the 21st century, stem cells have been identified in the human vagina [4]. Stem cells are undifferentiated cellular elements that actively contribute to the tissue regeneration when properly stimulated [5]. This particular cell population is responsible for vagina regenerative capability [3]. Nevertheless this regeneration property could be seriously counteracted by several kind of pathogen infections, affecting vaginal milieu [6].

In physiological conditions, the vagina is characterized by a complex and dynamic microbiome that continuously undergoes variations during woman’s life and menstrual cycle, actively contributing to reproductive tract health. In particular, colonization of the vagina by Lactobacilli (*Lactobacillus* spp.), during the reproductive age, is currently accepted as a biomarker of a healthy status [7]. The acidic pH of vaginal fluids (3.5–4.5) is considered suitable for Lactobacilli growth, representing a protective factor against pathogen proliferation, and therefore allows a healthy vaginal flora balance. Indeed, the majority of vaginal infections have been associated to vaginal milieu disorders [8].

Vaginal infections affect millions of women annually worldwide [9]. Different surveys estimated that over 70% of adult women have had a vaginal problem and have used vaginal products to treat infections. The commonest causes of vaginitis are vaginal bacteriosis (40–50% of cases), aerobic vaginitis (5–10%), vulvovaginal candidiasis (20–25%), and trichomoniasis (15–20%) [10].

Among vaginal infections, those caused by aerobic bacteria as *Escherichia coli* and *Staphylococcus aureus*, are named aerobic vaginitis, with a prevalence range from 5% to 10% in non-pregnant women [11,12]. This condition is featured by an inflammatory state and lack of Lactobacilli in vaginal milieu. Symptoms associated with aerobic vaginitis include redness of the epithelium, yellow discharge and dyspareunia [13].

The second most prevalent vaginal infection, vulvovaginal candidiasis, is the commonest clinical disease caused by Candida (*Candida* spp.) Approximately 70–75% of sexually active women have had at least one episode of Candida infection in their lifetime [14]. The commonest symptoms associated with Candida infections are vaginal irritation and discharge, vulvar burning, pruritus and swelling. Dyspareunia and dysuria and slight bleeding from fissures and excoriations could also be observed [15].

*Trichomonas vaginalis* is responsible for the commonest non-viral sexually transmitted infection, with an estimated 156 million cases every year worldwide [16]. Up to 80% of infected women are asymptomatic and the infection can persist for several months [17]. When present, symptoms include diffuse, malodorous, yellow-green, thin, watery vaginal discharge with pH > 4.5 and vulvar irritation [18]. *Trichomonas vaginalis* infection is related to preterm labor, low birth weight, prostate and cervical cancer and acquisition of human immunodeficiency virus [19].

Therapeutic agents are used to control and relieve the signs and symptoms and to reduce the amount of microorganisms. Aerobic vaginitis treatment is based on antibiotics against the pathogens responsible for these conditions. However, a standard treatment is still not available [11]. Vulvovaginal candidiasis is usually treated very effectively with azoles, including short course topical treatments [20]. Nevertheless, the treatment of vaginal infections in pregnancy indicates that the antibiotic treatment repairs the infection but does not completely reduce the risk of miscarriage and preterm delivery [21,22]. Moreover, the first-choice antimicrobials used to treat *Trichomonas vaginalis* infections are metronidazole and tinidazole (approved in USA), which are orally administered [23].

The limited number of therapeutic options available, together with the increase in microorganism drug-resistance, generated the need to research novel antimicrobials. The recent emerging trends concerning the use of natural products for medical treatment, improved the interest of researchers on ethnopharmacology and plant derived products fields [24].

Myrtle (*Myrtus communis* L.) is a bush widespread in Sardinia, commonly used in folk medicine. Phyto-derivatives can be obtained from three different parts of the plant: fruit, seeds and leaves. In particular, leaves and fruit decoctions were used in folk medicine to wash wounds, for vaginal lavage. Previous in vitro studies revealed that ethanol extracts, aqueous extracts and essential oils from myrtle exhibited antioxidant, antiseptic, anti-inflammatory and antiaging properties [25,26].

The usefulness of drugs for vaginal trait could be accomplished by novel specific strategies to enhance definite features, as controlled delivery, solubility and distribution in the target area, and also to promote a healthy environment by restoring features as vaginal pH, fluid and microflora [27].

The advent of pharmaceutical nanotechnology and nanomedicine also disclosed novel pharmacotherapeutic approaches for vaginal drug delivery [28]. Different nanotechnology-based systems have been extensively investigated for vaginal use, in particular for developing microbicides targeted on preventing sexual HIV transmission. Nanofibers are a novel dosage form for intravaginal controlled drug delivery that offer significant flexibility in product attributes to meet different needs [28].

In this study we fabricated novel nanodevices comprising Polycaprolactone (PCL) with Gelatin nanofibers, to deliver three natural extracts from myrtle leaves, seeds and fruit.

The aim of this study was to employ these nanodevices in order to evaluate their in vitro capability to inhibit the proliferation of different microorganisms implicated in vaginal infections. Nevertheless, considering the side effects of the main used treatments, we also evaluated the toxicity of the nanodevices against the main representative cell populations of the vagina and the surrounding area.

## 2. Results

### 2.1. Identification and Quantification of Phenolic Compounds

A total of 12 phenolic compounds, including two phenolic acids, six flavonols, and four anthocyanins, were identified from peel + pulp (fruit), seeds and leaf samples (Table 1). Among phenolic acids, gallic and ellagic acid were identified. In fruit and seeds samples, ellagic acid was the most dominant phenolic acid with a concentration of 450.3 ± 20.5 and 1050.3 ± 112.8 mg/kg_DW_, respectively. The contents of gallic acid varied from 353.4 ± 22.2 to 797.0 ± 60.5 mg/kg_DW_ in fruit and seeds, respectively. The gallic acid was the only phenolic acid identified in the leaves samples (1199.3 ± 70.4 mg/kg_DW_). Compared with other Myrtus parts, leaves contained higher flavonols concentrations.

Among these, myricetin-3-O-rhamnoside was the most abundant compound with a 3902.9 ± 117.7 mg/kg_DW,_ followed by myricetin-3-O-galactoside (1926.4 ± 6.2 mg/kg_DW_). Other identified flavonols were quercetin-3-O-glucoside, quercetin-3-O-rhamnoside, quercetin 3-O-galactoside and vitexin with a content ranging from 85.9 ± 3.0 mg/kg_DW_ to 280.0 ± 13.5 mg/kg_DW_ for quercetin 3-O-galactoside and vitexin, respectively. Flavonols were also found in seeds samples where quercetin-3-O-rhamnoside shows the higher concentration (160.6 ± 14.5 mg/kg_DW_). Myricetin-3-O-galactoside and quercetin-3-O-glucoside were not detected in seeds. As expected, anthocyanins compounds were found only in fruit samples, even in low concentrations. The contribution of anthocyanin to the phenolic compound amounts is, in fact, lower than 1%. Analysis by HPLC also revealed that a diverse range of hydrolysable tannins is present in fruit, seeds and leaves. This class of compounds is the most important for all samples analyzed, representing about 90% of phenolic compounds identified for seeds and leaves, and about 74% for fruit, respectively. Due to the lack of commercial standards, their concentration was calculated using gallic acid as reference. The highest value for these compounds has been recorded for seeds (25,016.2 ± 359.8 mg/kg_DW_), followed by leaves (21,858.3 ± 1099.3 mg/kg_DW_), and fruit (8612.0 ± 416.6 mg/kg_DW_). HPLC-DAD chromatograms of phenolic compounds can be found on Supplementary Materials.

### 2.2. Nanofibers Characterization

**Figure 1:**
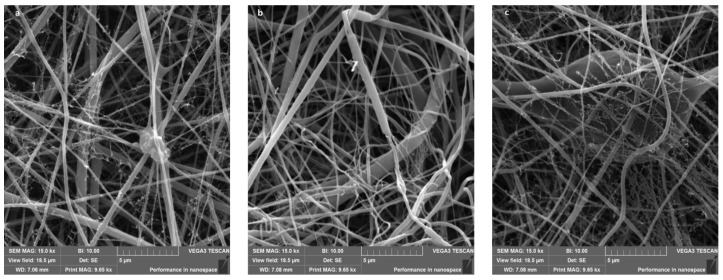
SEM microscope analysis showed the structure of nanofibers encapsulated with fruit (**a**), seeds (**b**) and leaves (**c**) extracts from myrtle.

Nanofibers made of PCL together with Gelatin were analyzed with scanning electronics microscopy (SEM) (Figure 1). Results were compared to nanofibers made with PCL only, in order to highlight the properties of the nanofibers created by adding Gelatin (Table 2). The soaked nanofibers were prepared using the white nanofibers (PCL and Gelatin), therefore the contact angle and diameters in this sample are also representative of all the soaked nanofibers. Comparing PCL and PCL plus Gelatin nanofibers, here we show that Gelatin addition to PCL electrospinning solution decreased the nanofiber diameter more than half-fold. Gelatin contains a lot of polar bonds. Therefore, Gelatin addition increases the electrical conductivity of the PCL electrospinning solution, resulting in the decrease in the nanofiber diameter.

Moreover, Gelatin addition to PCL electrospinning solution decreased the contact angle of the nanofibers. This event is related to the hydrophilic nature of Gelatin. Thus, the PCL nanofibers are hydrophobic, whereas the PCL and Gelatin nanofibers are hydrophilic. Interestingly PCL nanofibers and Gelatin with myrtle extracts exhibited hydrophobic features.

Nanofibers have a small diameter (Figure 1) making surface pressure a highly dominant phenomenon that significantly deteriorates the uptake and release of encapsulated molecules. Thus, a simple extract soaking into nanofiber core has only a low effect, and adsorbed molecules on the nanofiber surface are highly dominate over the extremely low extract concentration in the nanofiber core. Naturally, these adhered molecules would again be easily released once the extract concentration in the solution drops again.

A thorough study has been carried out through ATR-FTIR spectroscopy to better understand the details of nanofiber-extract interactions in the case of soaked and encapsulated nanocomposites, and to evidence differences in the two kinds of nanofibers, if any exist.

**Figure 2:**
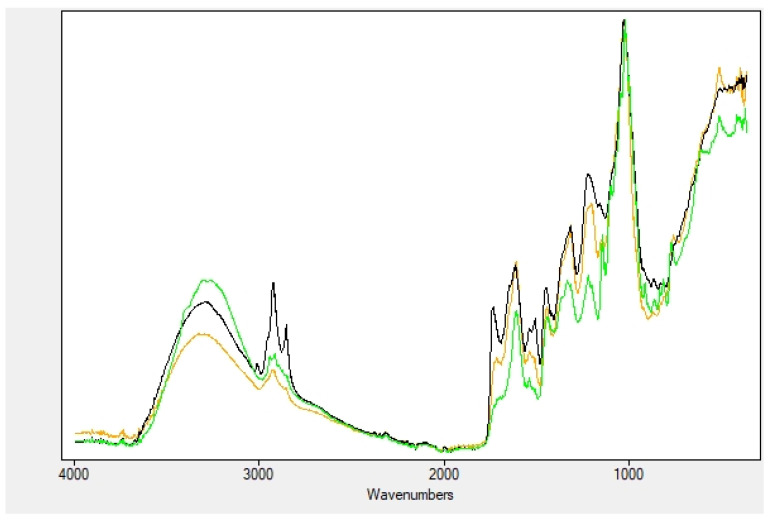
IR: comparison of myrtle dry extracts: fruits (green), leaves (orange) and seeds (black).

Dry extracts of myrtle fruits, leaves and seeds show very similar FTIR spectra (Figure 2). Despite their composition, they may differ in terms of the specific compounds present in the extracts (*vide supra*), and they share the same functional groups related to aromatic and secondary alcohols (phenols and glucide moieties), ethers, ketones, hydrocarbons. Terpenoids, lipids, proteins, enzymes and polysaccharides are also present [29,30].

Thus, their FTIR spectra are rather complicated. What is easily distinguishable is the very broad OH stretching band around 3300 cm^−1^, together with the intense and broad band around 1030 cm^−1^, ascribed to the C-O stretching vibrations of all the secondary alcohol groups on the different glycosides present in the extracts. Other C-O stretching vibration absorption bands at wavenumbers 1400–1300 and 1230–1140 cm^−1^ are attributed to the flavonoid compounds. Bands at around 1230, 1200, and 1155 cm^−1^ can be ascribed to the C–O stretching in the aryl ether rings, the C-O stretching in phenols, and the C–CO–C stretch and bending in ketones, respectively. The OH bending occurs at around 1350 cm^−1^. The C-H stretching vibration of the hydrocarbon portions are found in the region of 2900–2800 cm^−1^, while the C=C aromatic ring stretches are detectable at 1610, 1540, and 1510 cm^−1^. The broad and complex bands in the C=O stretching region (1760–1700 and 1680–1630 cm^−1^) are indicative of aldehyde, ketone and carboxylic groups of the former, and amide groups of the latter, respectively. The other bands down in the fingerprint region cannot be attributed with certainty.

**Figure 3:**
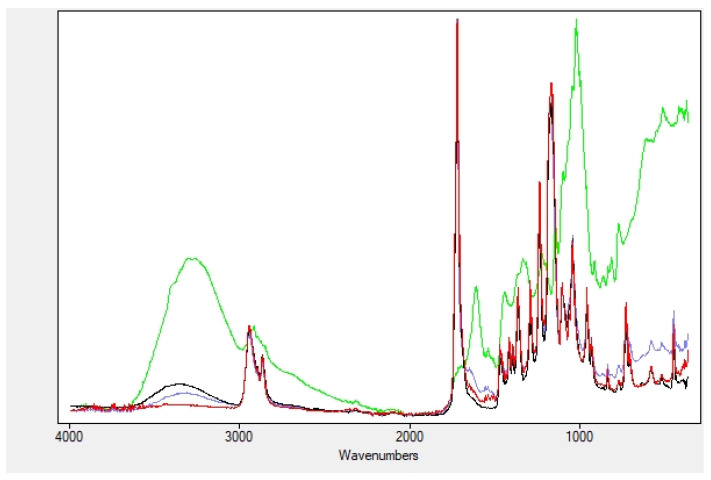
Comparison dry extracts of fruits (**green**), soaked nanofibers (**black**), encapsulated nanofibers (**blue**) and “naked” nanofibers (**red**).

The FTIR spectrum of the PCL + gelatin nanofibers is reported in Figure 3 (red line) and is analogous to that of the commercially available nanocomposite, as described in the literature (see for instance Biological behavior study of gelatin coated PCL nanofiberous electrospun scaffolds using fibroblasts [31]). The bands between 2940 and 2860 cm^−1^ are due to the aliphatic C-H stretching (CH_2_ symmetric and asymmetric stretch vibration), the ester C=O stretching lies at 1724 cm^−1^, while amide I and amide II vibrations of gelatin can be identified in the multiple bands centered at 1648 cm^−1^ and 1537 cm^−1^, respectively, corresponding to the stretching vibrations of C-O bond, and of N-H bond and stretching of C-N bonds, respectively. The amide I band at 1648 cm^−1^ can be attributed to both an α-helix and random coil conformation of gelatin.

**Figure 4:**
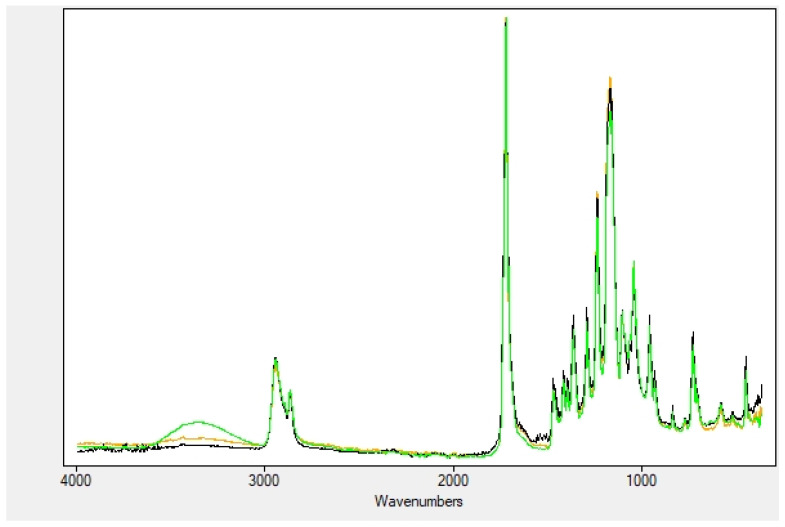
Comparison of soaked nanofibers: fruits (**green**), leaves (**orange**) and seeds (**black**).

Figure 4 reports the superposition of the nanofibers soaked with fruit, leaf and seed extracts. The predominance of the nanofiber bands, probably due to the fact that the amount of plant biomolecules is largely exceeded by the nanocomposite material, covers those of the extracts. What is interesting to note, instead, is the presence of the OH stretching vibration, which was obviously not observed in the nanofibers alone. Moreover, the position of this band, previously centered at 3300 cm^−1^, has shifted to higher wavenumbers compared to the free extracts and is now lying at 3360 cm^−1^. This blue shift may be due to hydrogen bond interaction between the OH groups of the myrtle biomolecules and the polar groups on the gelatin peptides (carboxyl, amide and amine groups, either on the backbone and the side chains). The amide bands of gelatin, on the other hand, have broadened and decreased in intensity. Examination of the spectrum shows a slight red shift, with the broad bands now centered at 1644 and 1530 cm^−1^, respectively), again a probable consequence of the hydrogen bonds with the OH groups in the extracts (Table 3).

**Figure 5:**
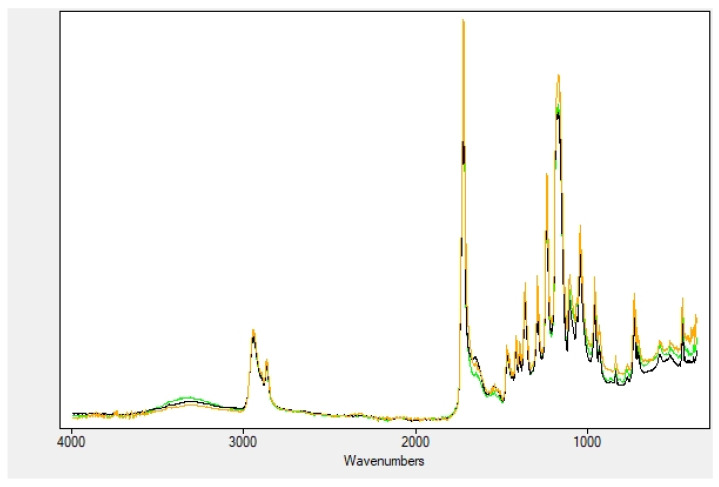
Comparison of encapsulated nanofibers: fruits (**green**), leaves (**orange**) and seeds (**black**).

Figure 5 reports the superposition of the nanofibers with encapsulated extracts of myrtle fruits, leaves and seeds. Here the shift of the OH band shows a different trend, since in this case it moved to lower wavenumbers (3290 cm^−1^). The amide I and amide II complex bands, on the other hand, are now centered at 1657 and 1545 cm^−1^, respectively, with a blue shift. The rest of the spectra seems to be unaffected by the encapsulation of the extracts.

Figure 3 reports the comparison of dry extracts, “blank” nanofibers, nanofibers with soaked extracts and nanofibers with encapsulated extracts, only for the myrtle fruit (but those for the leaf and seed extracts, not shown, indicate the same trend), to highlight this behavior. The shift of the OH and amide bands is clearly observable both in the soaked and the encapsulated nanofibers.

The difference in the position of the OH and amide bands that was observed in the two kinds of nanocomposites seems to be involved in the formation of hydrogen bonds among the OH groups of the flavonoids/anthocyanins/polyphenolic compounds of the plants and the gelatin portion of the nanofibers. The nature of these interactions, surely different in the two cases, determines the extent and direction of the band shift observed, and is a clear indication that the biomolecules of the extracts have different “intimate contacts” with the nanofibers in case they are soaked or encapsulated.

### 2.3. Nanofibers with Myrtle Extracts Exert Selective Antibacterial and Antifungal Action

Six different combinations of nanofibers, both encapsulated or soaked with myrtle extracts (0.2 mg/disc), were tested at two different concentrations as described in Table 4.

The antimicrobial activity was evaluated against Escherichia coli, Staphylococcus aureus, Lactobacillus acidophilus, Candida parapsilosis, Candida krusei Candida albicans, Candida glabrata, and Candida kefir.

**Figure 6:**
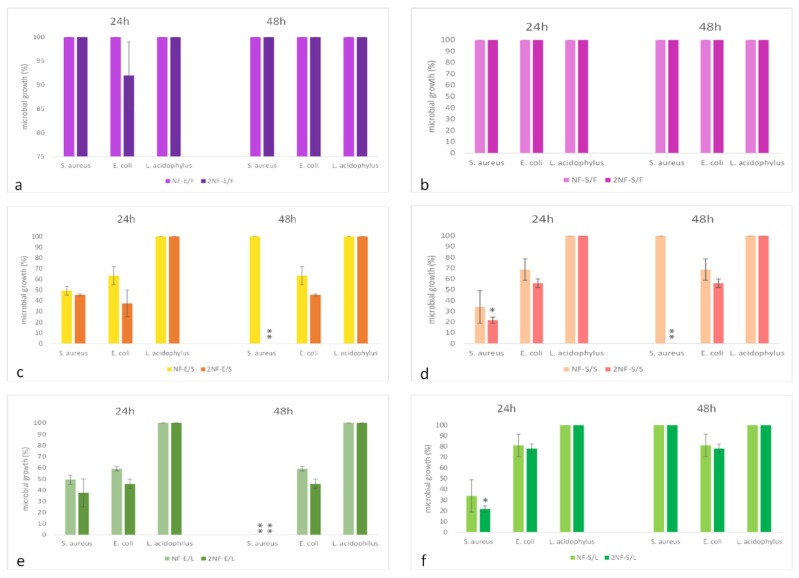
Effects of nanofibers containing leaves, seeds and myrtle fruit extracts soaked (right graphs **a**, **c** and **e**) or encapsulated (left graphs **b**, **d** and **f**) against bacteria, after 24 h and 48 h of incubation. Data are expressed as means percentage ± standard deviations compared with control (100%). * *p* ≤ 0.05; ** *p* ≤ 0.01.

**Figure 7:**
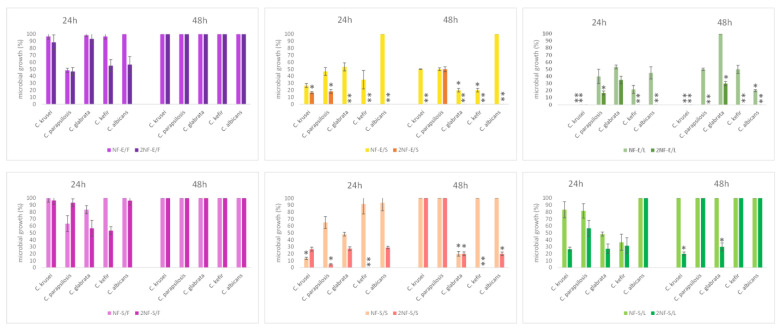
Effects of nanofibers containing leaves, seeds and myrtle fruit extracts soaked or encapsulated against *Candida* spp., after 24 h and 48 h of incubation. Data are expressed as means percentage ± standard deviations compared with control (100%). * *p* ≤ 0.05; ** *p* ≤ 0.01.

Results are showed in detail in Figure 6 and Figure 7.

The treatments NF-E/F, 2NF-E/F, NF-S/F, 2NF-S/F, consisting of nanofibers soaked or encapsulated with fruit extract from myrtle, do not show significant antimicrobial activity on the microorganisms tested. In general, the more effective treatments 2NF-E/L and 2NF-S/S comprise two nanofiber discs encapsulated with leaves extract or two nanofibers discs soaked with seeds extract.

The treatments 2NF-E/S and 2NF-E/L are the most effective against *E. coli* with a decrease of 60% of bacterial growth, as compared to untreated control. Moreover, the treatment 2NF-S/S showed a decrease of 40% of the bacterial growth. Other treatments tested do not show a significant activity against *E. coli*. Results obtained after 24 h were also confirmed after 48 h.

*S. aureus* growth was highly inhibited by treatment 2NF-E/L and 2NF-S/S (80% reduction) after 24 h of treatment (Figure 6), while a decrease of 50% and 60% of bacterial growth was observed after 24 h of co-incubation with NF-E/L and 2NF-E/S nanofibers, respectively. Interestingly, *S. aureaus* growth was completely inhibited after 48 h.

The treatment 2NF-E/L (two discs of nanofibers encapsulated with leaves extracts) completely inhibits the growth of *C. parapsilosis*, *C. krusei*, *C. albicans*, and *C. kefir* at 24 h. The NF-E/L treatment against *C. glabrata* had a moderate inhibitory effect at 24 h, which disappears at 48 h of incubation (Figure 7).

The tests comprising treatment 2NF-S/S totally inhibited growth of *C. glabrata* and *C. kefir* after 24 h. The same nanofibers caused a growth decrease after 24 h of treatment for *C. parapsilosis*, *C. krusei* and *C. albicans* (80%).

Interestingly, no type of nanofiber with the extracts exerted any effect on the growth of *L. acidophilus* (growth 100%; Figure 6).

Empty (blank) nanofibers and soaked nanofibers with ethanol did not show antibacterial activity against bacteria and yeast.

### 2.4. Nanofibers with Myrtle Extracts Counteract Trichomonas vaginalis Strain Viability

**Figure 8:**
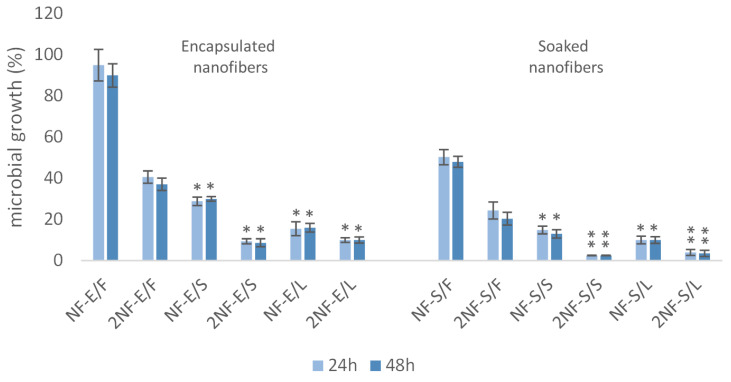
Effects of nanofibers containing leaves, seeds and myrtle fruit extracts soaked or encapsulated against Trichomonas vaginalis G3, after 24 h and 48 h of incubation. Data are expressed as means percentage ± standard deviations compared with control (100%). * *p* ≤ 0.05; ** *p* ≤ 0.01.

*Trichomonas vaginalis* G3 was evaluated in vitro to verify the cytotoxic effect of myrtle extracts combined with nanofibers. Antitrichomonal activities were tested by incubating nanofiber containing extracts from leaves, seeds and fruit soaked or encapsulated, for 24 and 48 h with protozoa. Nanofibers without extracts have been used as negative control. Our results show that antitrichomonal activities of the different nanofibers tested were concentration-dependent (Figure 8).

Figure 8 lists the average percentage of inhibition elicited by the nanofibers containing leaves, seeds and myrtle fruit, soaked or encapsulated on *T. vaginalis* G3 under the described test conditions.

The most effective treatments against the reference strain G3, with a 95% reduction in cell viability, are 2NF-S/S and 2NF-S/L, those with two discs of nanofibers soaked using seed extracts and leaves extracts.

In particular, treatments 2NF-S/S and 2NF-S/L were able to exert a 95% decrease in G3 strain cell viability (Figure 8). A similar effect could be observed for treatments 2NF-E/S, 2NF-E/L, NF-S/S and NF-S/L (90% decrease in cell viability).

Results achieved after 48 h of treatment do not show significant differences, confirming the results obtained after 24 h.

### 2.5. Nanofibers with Myrtle Extracts Does Not Affect Eukaryotic Cell Viability

**Figure 9:**
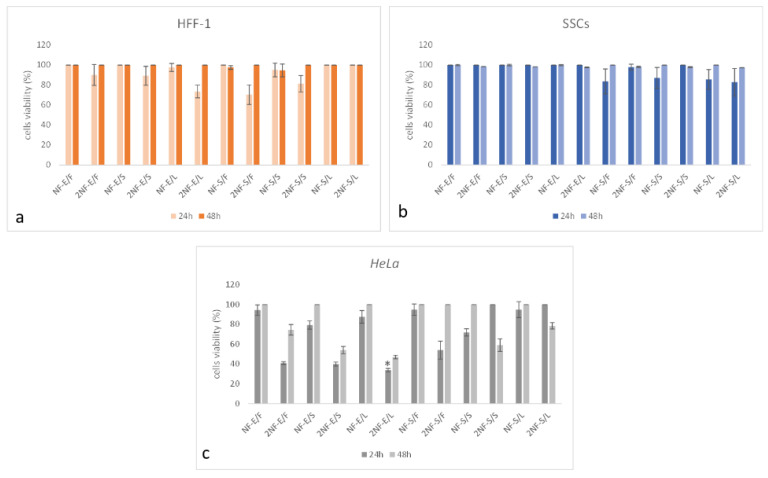
Results of MTT assay of HFF-1 (**a**), SSCs (**b**) and HeLa cells (**c**). Graphs represent cell viability after 24 or 48 h of treatment with the six treatments tested. Data are expressed as means percentage ± standard deviations compared with control (100%). * *p* ≤ 0.05.

Cells susceptibility to myrtle extracts combined with PCL nanofibers was evaluated by culturing *HeLa*, HFF1 and SSCs cells for 24 h and 48 h under the different experimental condition described in Table 1. As shown in Figure 9, treatments do not affect HFF1 and SSCs cells, neither 24 h nor 48 h after. A moderate toxicity could be observed on *HeLa* cells (50% decrease in cell viability) after 48 h of treatments 2NF-E/S, and 2NF-E/L. On the other hand, other treatments (NF-E/F, 2NF-E/F, NF-E/S, NF-E/L, NF-S/F, 2NF-S/F, NF-S/S, NF-S/L) produced an early cytotoxic effect that disappears after 48 h (Figure 9c).

## 3. Discussion

In recent years the need of novel antimicrobial treatments has emerged to counteract multiresistant microorganisms and to meet market expectations concerning the use of natural substances [32]. Nanofibers are innovative devices already successfully tested for vaginal drug delivery. Vaginal infections are a reproductive health problem worldwide affecting a high number of women at reproductive age and associated to different discomfort and complications reducing women’s quality of life [33]. The presence of the local flora, populated by *Lactobacillus* spp. allow the maintenance of physiological conditions [7]. Indeed, recent studies show the role of Lactobacilli in accelerating re-epithelialization in vaginal epithelial cells, suggesting a protective role of the milieu in defending the vaginal epithelium from damages and risk of pathogenic infections [34].

Berendika et al. in 2022 demonstrated that some components of myrtle extracts as Polyphenolic and flavonoid compounds, including quercetin and proanthocyanidin, promote Lactobacilli growth [35].

Our results showing that nanofibers made with PCL and Gelatin, encapsulated or soaked with extracts of seeds, leaves and fruit of myrtle, did not affect *Lactobacillus acidophilus* behavior. This result does not dispute with the previous findings of Berendika, since in our experiments we did not observe any growth inhibition, although there was no evidence of a promoting-growth effect on *L. acidophilus.*

On the other hand, we demonstrate that some combinations of nanofibers with myrtle extracts bear an antimicrobial action against several pathogens and opportunistic microorganisms. Experiments reveal that the combinations of two nanofiber discs encapsulated with leaves extracts or two nanofiber discs soaked with seeds extracts (treatments 2NF-E/L and 2NF-S/S) are the most effective against the microorganisms tested in this study. In particular our experiments reveal a complete inhibition of *S. aureus* and all the tested *Candida* species after 48 h of incubation with nanofibers encapsulated with myrtle leaves extracts (treatment 2NF-E/L).

Several studies demonstrate that antimicrobial activity of myrtle leaves extracts is due to the presence of phenolic compounds as flavenols (quercetin glycosides and myricetin) and derivatives of galoyl or to the presence of polyphenols [36].

According to the literature, the results obtained here show that the extracts of seeds and leaves of myrtle exhibit an antimicrobial activity. Within this context their composition is rich in the previously defined components, already described for their antimicrobial effect. On the contrary, fruit extracts do not show significant antimicrobial activity, probably due to the absence of these compounds (Table 1).

All the treatments used in this study showed a moderate effect against the Gram-negative *E. coli* compared to the Gram-positive *S. aureus.* This result could be ascribed to the different composition and structure of the cell wall of these microorganisms. Actually other authors recognized that Gram-negative bacteria are more resistant to plant-based extracts [37].

The anti-trichomonal effect of phyto-derivates [38,39] has already been described in the literature. Our results demonstrate the efficiency of nanofibers differently combined with myrtle extracts (2NF-E/S, 2NF-E/L, NF-S/S, 2NF-S/S, NF-S/L, and 2NF-S/L; Figure 8) against the reference strain G3. Nevertheless, the wide variability in the intrinsic characteristics of *T. vaginalis* strains, and the symbiosis with *Mycoplama hominis* [40,41], raises the need for other studies to also evaluate other strains besides G3.

On the other hand, an essential condition that new approaches able to fight infections should bear is the ability to preserve the physiological health of the environment and cell types involved in repairing damaged tissues. In particular, stem cells have an important role in tissue regeneration [5]. In the present study, stem cells, fibroblast and human epithelial cells were analyzed. These cells are present in this area allowing the hydration of the vaginal mucosa and tissue repair [2].

Our results show that nanofibers of PCL and Gelatin with myrtle extracts do not affect fibroblast (HFF-1) and skin stem cells (SSCs) viability. However, human epithelial cell (*HeLa*) viability was quite inhibited after the treatment. The moderate cytotoxicity against *HeLa* cells could be an interesting starting point to investigate in more detail other effects of these nanomaterials. Notably, *HeLa* is the only tumoral cell line tested by us, and is the only cell population damaged after treatment.

The selective capability of PCL and Gelatin nanofibers with myrtle do not influence human cell viability can be explained by myrtle plant features, comprising compounds bearing, not only natural antimicrobial, but also anti-inflammatory and regenerative properties [39]. Within this context, many studies show the ability of myrtle extracts or essential oils to counteract the growth of *Streptococcus pneumoniae*, *Streptococcus pyogenes*, *Streptococcus agalactiae*, *Listeria monocytogenes*, *Escherichia coli*, *Proteus vulgaris*, *Pseudomonas aeruginosa*, while antifungal activity of myrtle essential oil was tested against different *Candida* species. Moreover, the anti-inflammatory properties of myrtle could represent a valuable tool to prevent or treat the inflammatory conditions related to infections: myrtle, is an important natural source of antioxidants, important for reducing oxidative stress in human cells. Nevertheless, it was also observed that there was a pro-apoptotic activity of the plant in cancer cells in vitro [42].

Another feature highlighted by our studies is that nanofibers made of PCL and Gelatin with myrtle extracts are hydrophobic. Cell adhesion, communication, and proliferation can be effectively controlled, acting on chemical and physical features of scaffolds [43].

Hydrophobicity of these novel nanofibers fabricated here, could be a disadvantage to produce scaffolds. In fact, cells prefer moderate hydrophilic surfaces to growth as hydrophobic surfaces repulse water molecules, a key factor for cell life and adhesion.

However, the use of these nanofibers as devices for the controlled release of molecules could hypothetically justify the major ability of PCL nanofibers to counteract Gram-positive microorganisms better than Gram-negative microorganisms. The capability of hydrophobic surfaces to suppress protein absorption undergoing denaturation processes, allow the protein to bind to the material surface, exposing the internal hydrophobic residues [43]. This process is less effective in Gram negatives that protected proteins with the external membrane.

Nevertheless, our results highlight no antimicrobial action of the nanofibers both soaked and encapsulated with the myrtle fruit. On the other hand, nanofibers encapsulated or soaked with leaves or seeds exhibited an antimicrobial activity. However, it is not clear why the soaked nanofibers work better in some cases and encapsulated ones in others. Presumably the different effectiveness could be related to the bonds that are formed between the different components of the extracts and the material composing the nanofibers or, in the case of the soaked ones, to the interactions of molecules with alcohol and its evaporation. Future studies are needed to elucidate the interactions between extracts and matrices and the mechanisms of action of the compounds against microorganisms.

## 4. Materials and Methods

### 4.1. Plant Material Preparation and Characterization

#### 4.1.1. Chemicals

Acetonitrile was HPLC grade and was purchased from Sigma (Merck Life Science S.r.l., Milano, Italy). Orthophosphoric acid (ACS ISO, for analysis, 85%) and ethanol (ACS-Reag. Ph.Eur. Supelco^®^, Milan, Italy) were purchased from Carlo Erba..Water was distilled and filtered through a Milli-Q apparatus (Millipore, Milan, Italy).

Standards of gallic acid and ellagic acid were purchased from Sigma (Merk Life Science S.r.l., Milan, Italy). Standards of myricetin-3-O-galactoside, myricetin-3-O-rhamnoside, quercetin-3-O-glucoside, quercetin-3-O-rhamnoside, quercetin 3-O-galactoside, vitexin, cyanidin 3-O-glucoside, petunidin 3-O-glucoside, peonidin 3-O-glucoside and malvidin 3-O-glucoside were purchased from Extrasynthese (Lyon, France).

#### 4.1.2. Plant Material

Myrtle berries byproducts were kindly provided by a local farm, and leaves were randomly harvested from June to July at the Experimental Station ‘Antonio Milella’ of the University of Sassari in Fenosu (Oristano, Sardinia, Italy). Samples were frozen in liquid nitrogen, freeze-dried (Edwards Lyophiliser, Bolton, UK), and stored in the dark at room temperature and under low relative humidity (RH) conditions until the preparation of the extracts. After lyophilization, berries were divided into two parts: peel + pulp and seeds.

#### 4.1.3. Preparation of Extracts

Extraction of phenolic compounds was determined according to the previous methods, opportunely modified, developed by Fadda et al. [44] and by Sarais et al. [45] for leaves and berries, respectively. Briefly, dried samples were thinly pulverized in a home style coffee grinder just before preparation of the extracts, mixed thoroughly and split up into three replicates. An aliquot of solid sample was extracted with an ethanol/water solution (70% EtOH) with solid:solvent ratio 1:20 g/mL. The extraction was conducted in an ultrasonic bath for 60 min, where the temperature was maintained at a constant level by the circulation of water in an outer jacket connected to a thermostat. The extract was centrifuged at 4000 rpm for 60 min at 20 °C and diluted 1:50 (*v*/*v*) with 0.22 M phosphoric acid in water before injection into the chromatographic system.

#### 4.1.4. HPLC-DAD Analysis of Phenolic Compounds

An Agilent 1100 system consisting of a G1311A quaternary pump, a G1313A rheodyne injector, a G1316A thermostated column compartment, a G1322A degasser, and coupled with a DAD detector UV 6000 (Thermo Finnigan, Milan, Italy) was employed to separate and quantify phenolic compounds. Analyses were performed using a Kinetex column (5u, C18, 100 A; Phenomenex, Torrance, CA, USA), eluted with mobile phase A (acetonitrile) and B (H_2_O with 0.22 M phosphoric acid). A linear gradient program at a flow rate of 0.4 mL/min was used: 0–30 min from 5 to 10% (A); 30–35 min from 10 to 15% (A); 35–70 min from 15 to 30% (A); 70–100 min from 30 to 90% (A); then to 100% (A) up to 120 min. A post-time of 20 min was used to allow the column to equilibrate before the next sample injection. Detection was carried out at 280 nm for gallic acid and hydrolysable tannins, 360 nm for flavonols and ellagic acid, and 520 nm for anthocyanins. Extracts were directly injected onto an HPLC after dilution with phosphoric acid 0.22 M. The results were expressed as mg/kg of dry weight. All tests were carried out in triplicates, and the standard deviation was calculated for all data. A quantitative determination was carried out using a multiple-point external standard method created by preparing a set of standard solutions with known concentrations of each analyte (the concentration range used to construct the calibration lines was 0.02–20 mg L). Calibration curves were created by plotting the peak area versus the nominal concentration of the analytes.

### 4.2. Nanofibers Fabrication and Functionalization

#### 4.2.1. Electrospinning of Nanofibers

Nanofibers were prepared using electrospinning of PCL together with Gelatin blend. Firstly, Gelatin (Merck, KGaA, Darmstadt, Germany) was dissolved in a solution of acetic and formic acid (both Sigma-Aldrich, St. Louis, MO, USA) (1:1 *v*/*v*) using stirring at room temperature. After Gelatin dissolving, PCL (MW 45 kDa, Sigma-Aldrich, USA) was added and dissolved in the Gelatin solution in acids (PCL/Gelatin 9:1). The 18% *w*/*w* PCL and Gelatin blend was electrospun in a spinner, a Nanospider (Elmarco, Svàrovskà, Czech Republic), with a needleless wire electrode, a wire collector covered with a PP non-woven textile (Pegas, Czech Republic) and the electric field intensity 0.4 kV/cm for several hours.

Dried extracts of myrtle (leaves, fruit and seeds) were grinded in liquid nitrogen for 30 min using a cryogenic grinder (Freezer/Mill 6870, SPEX SamplePrep, Metuchen, New York, USA) to minimalize the size of particles for encapsulation into nanofibers. A total of 1.73 g of grinded extract was added to 25 g of 18% *w*/*w* PCL and Gelatin blend (9:1 *w*/*w*) in acetic acid.

Blank samples were prepared with the same method, but without encapsulating the extracts.

#### 4.2.2. Nanofibers Characterization

Nanofibers morphology was observed by scanning electron microscopy (VEGA 3 SBU, Tescan). Before SEM analysis, all nanofibers samples were sputtered with thin gold layer by a rotatory pumped coater (Q 150R S, Quorum). The average diameter was measured for 100 randomly selected fibers using Tescan software (Tescan, Brno-Kohoutovice, Czech Republic).

The contact angle was measured by a self-made device with a 5 Mpix camera with 10× magnification. A small droplet (3 µL) of distilled water was placed at the top of a sample using a pipette and photos were taken with the device. Captured photos were analyzed by ImageJ software with plugin for contact angle measurement (n = 10 for each type of nanofiber). Contact angle was measured at 24 °C.

Infrared spectroscopy was performed by IR spectrometer (IRAffinity-1, Shimadzu) with ZnSe crystal, resolution 8 1/cm, range 4000–5501/cm (n = 100 scans per sample) and using FTIR-ATR (attenuated total reflection) method.

#### 4.2.3. Preparation of Nanofibers Samples

To perform the experiments nanofibers towels were sterilized with ethylene oxide and cut in 6 mm diameter discs. To check the thickness of the nanofibers, 30 nanofibers discs were measured. Mean thickness was estimated to be 103 ± 12.9 μm. To compare the effect of encapsulation and the use of the extracts in combination with the nanofiber causing weak interactions between extracts and the porous surfaces of the material, blank samples were soaked with the same extracts powder resuspended in 70 % ethanol at the same concentration. Each nanofiber disc was soaked with 10 µL of extract (20 mg/mL) and let dry for almost 30 min before use to evaporate ethanol from the fibers. The experimental conditions tested are illustrated in Table 4.

### 4.3. Microorganisms Selected and Culturing Conditions

Escherichia coli (ATCC 25922), Staphylococcus aureus (ATCC 29213), five different *Candida* spp. (Candida albicans, Candida Kefyr, Candida Krusei, Candida glabrata, Candida parapsilosis) and Lactobacillus acidophilus obtained by clinical vaginal samples, were grown in Luria–Bertani (LB) broth, at 37 °C in constant agitation.

*Trichomonas vaginalis* reference strain G3 was cultured by daily passages in Diamond’s medium supplemented with 10% heat-inactivated bovine serum, in 5% carbon dioxide atmosphere, at 37 °C [16].

### 4.4. Human Cell Populations Choice and Culturing Conditions

In vitro experiments were assessed by using Human Foreskin Fibroblast 1 cell line (HFF-1), Skin stem cells (SSCs), and *HeLa* cell line.

SSCs were collected and isolated from the Dermatological Clinic, Department of Medical, Surgical and Experimental Sciences, in Sassari as described before [46] after consent and ethical committee (Ethical Clearance N. 0021565/2018, 22/03/2018-Commissione Etica CNR). SSCs and HFF-1 cell line were cultured in Dulbecco modified eagle medium (D-MEM, Gibco) supplemented with 10% fetal bovine serum, 100 I.U./mL penicillin (Euroclone, Milano, Italy), and 100 μg/mL streptomycin (Euroclone, Milano, Italy), and L-glutamine (Euroclone, Milano, Italy), at 37 °C in a 5% CO_2_ atmosphere.

*HeLa* were cultured in RPMI 1640 medium (Sigma Chemical Co., St. Louis, MO, USA) supplemented with 10% inactivated fetal bovine serum, 100 I.U./mL penicillin and 100 μg/mL streptomycin, at 37 °C in a 5% CO_2_ atmosphere.

### 4.5. In Vitro Antibacterial and Anticandidal Activity of Nanofibers with Myrtle Extracts

The antibacterial and anticandidal activity of the nanofibers was evaluated against *C. albicans*, *C. glabrata*, *C. krusei*, *C. kefir*, *C. parapsilosis*, *E. coli*, *S. aureus*, and *L. acidophilus*, *E. coli* and *S. aureus* were chosen to represent Gram-positive and Gram-negative bacteria responsible for aerobic vaginitis. Briefly, 200 μL of LB broth containing 150 mid-logarithmic phase *E. coli* cells or 250 cells of the other microorganisms were seeded for well, in a 96-wells plate, with one or two 6 mm diameter nanofiber discs, as described above in Table 4. LB broth was used as growth controls and nanofibers blank samples were used as negative control. After 24 and 48 h of incubation with nanofibers at 37 °C, growth inhibition of the five *Candida* species isolates was verified by microscopic observation. Bacterial growth was measured by spectrophotometer at 600 nm. Results were expressed as percentage of viability referred to negative control.

### 4.6. In Vitro Anti Trichomonas vaginalis Activity of Nanofibers with Myrtle Extracts

*Trichomonas vaginalis* G3 in exponential growth phase were seeded into a 96-wells plate at the concentration of 2 × 10^4^ cells/well. Protozoa were incubated at 37 °C, in 200 µL of complete Diamond’s medium with one or two, 6 mm diameter nanofiber discs, as described above. Complete Diamond’s medium was used as growth controls and nanofibers blank samples were used as negative control.

Results obtained were assessed under the optical microscope after 24 and 48 h of incubation at 37° with the treatment, 10 µL of trichomonad culture were harvested from each well, and viable cells were counted using the Burker chamber. Results were expressed as percentage of viable cells compared with the negative control.

### 4.7. In Vitro Evaluation of Nanofibers Cytotoxicity on Human Cells HeLa, HFF-1 and SSCs

Cells were seeded into a 96-wells plate at the concentration of 15 × 10^3^ cells/well with the proper medium and let adhere overnight. Afterwards, the cells were treated with one or two 6 mm diameter nanofiber discs of each type for 24 or 48 h. The blank nanofibers represented the negative control. Cell viability was investigated by MTT (3-(4,5-dimethylthiazol-2-yl)-2,5-diphenyltetrazolium bromide) assay as described elsewhere [26]. Cell viability was detected by a plate reader (570 nm) and expressed as percentage of cell viability compared to control (100%).

### 4.8. Statistical Analysis

Each experiment was performed in triplicate and repeated three times. Statistical analyses were performed with ANOVA test. A *p* < 0.05 was considered significant.

## 5. Conclusions

The present study shows a well-defined capability of myrtle nanofibers to counteract microbial growth preserving the vaginal milieu, while complying resident cell populations in vitro. The use of nanofibers could facilitate the controlled release of myrtle active compounds, thus avoiding the use of excipients. Within this context, other authors described sensitive reactions that could be caused by excipients contained in 93% of medications [47]. Nanofibers enrolment could avoid the use of different excipients, contributing to the production of a safer product limiting the side effects associated to the treatments.

All together our results reveal that nanofibers of PCL Gelatin differently combined with myrtle extracts represent an interesting candidate for vaginal infection treatments.

## Figures and Tables

**Table 1:** Phenolic compounds concentrations in *Myrtus Communis* L. samples’ extracts (mg/kg_DW_ + SD, *n* = 3). ^($)^ Expressed as gallic acid equivalent.

Compound	λmax Uv-Vis (nm)	Concentration (mg/kg_DW_ ± SD; *n* = 3)		
		Peel + Pulp	Seeds	Leaves
Phenolic acids Gallic acid Ellagic acid Hydrolysable tannins ^($)^ Flavonols myricetin-3-O-galactoside myricetin-3-O-rhamnoside quercetin-3-O-glucoside quercetin-3-O-rhamnoside quercetin 3-O-galactoside vitexin Anthocyanins cyanidin 3-O-glucoside petunidin 3-O-glucoside peonidin 3-O-glucoside malvidin 3-O-glucoside	280 360 280+360 360 360 360 360 360 360 520 520 520 520	353.4 ± 22.2 450.3 ± 20.5 8612.0 ± 416.6 - - - - - - 2.8 ± 0.2 5.6 ± 0.6 2.5 ± 0.3 13.1 ± 0.4	797.0 ± 60.5 1050.3 ± 112.8 25,016.2 ± 359.8 - 49.7 ± 1.4 - 160.6 ± 14.5 31.0 ± 2.2 45.3 ± 3.1 - - - -	1199.3 ± 70.4 - 21,858.3 ± 1099.3 1926.4 ± 6.2 3902.9 ± 117.7 104.1 ± 4.6 192.0 ± 7.2 85.9 ± 3.0 280.0 ± 13.5 - - - -

**Table 2:** Shows measured values of average nanofiber diameter and contact angle.

Nanofibers	Average Diameter [nm]	Contact Angle
PCL	320 ± 190	129.2 ± 2.8°
PCL and Gelatin	129 ± 197	33.2 ± 3.5°
PCL and Gelatin encapsulated with leaves extract	104 ± 88	109.9 ± 6°
PCL and Gelatin encapsulated with seeds extract	147 ± 156	122.8 ± 5.7°
PCL and Gelatin encapsulated with fruit extract	166 ± 177	117.3 ± 5.8°

**Table 3:** Prominent band shift evidenced by FTIR analysis (wavenumbers are given in cm^−1^).

	OH Stretching	Amide I	Amide II
Extracts	3300		
NF		1648	1537
Soaked NF	3360	1644	1530
Encapsulated NF	3290	1657	1545

**Table 4:** List of treatments made combining PCL and Gelatin nanofibers with myrtle extracts.

Name of the Treatment	Treatment
NF-E/F	One 6 mm disc of nanofibers made of PCL with Gelatin encapsulated with myrtle fruits extract
2NF-E/F	Two 6 mm discs of nanofibers made of PCL with Gelatin encapsulated with myrtle fruits extract
NF-E/S	One 6 mm disc of nanofibers made of PCL with Gelatin encapsulated with myrtle seeds extract
2NF-E/S	Two 6 mm discs of nanofibers made of PCL with Gelatin encapsulated with myrtle seeds extract
NF-E/L	One 6 mm disc of nanofibers made of PCL with Gelatin encapsulated with myrtle leaves extract
2NF-E/L	Two 6 mm discs of nanofibers made of PCL with Gelatin encapsulated with myrtle leaves extract
NF-S/F	One 6 mm disc of nanofibers made of PCL with Gelatin soaked with myrtle fruit extract
2NF-S/F	Two 6 mm discs of nanofibers made of PCL with Gelatin soaked with myrtle fruit extract
NF-S/S	One 6 mm disc of nanofibers made of PCL with Gelatin soaked with myrtle seeds extract
2NF-S/S	Two 6 mm discs of nanofibers made of PCL with Gelatin soaked with myrtle seeds extract
NF-S/L	One 6 mm disc of nanofibers made of PCL with Gelatin soaked with myrtle leaves extract
2NF-S/L	Two 6 mm discs of nanofibers made of PCL with Gelatin soaked with myrtle leaves extract

## Data Availability

Not applicable.

## Supplementary Materials

The following supporting information can be downloaded at: https://www.mdpi.com/article/10.3390/plants11121577/s1, Supplementary material HPLC-DAD chromatograms of phenolic compounds in *Myrtus communis* L. sample’s extracts.
