# Supplementary material for: Myrtle-Functionalized Nanofibers Modulate Vaginal Cell Population Behavior While Counteracting Microbial Proliferation
Source: Plants (Basel). 2022 Jun 15;11(12):1577. doi: 10.3390/plants11121577 (PMC9227804; doi:10.3390/plants11121577)
Supplement: Supplementary file 1 [file plants-11-01577-s001.zip › plants-1678802-supplementary.pdf]

Supplementary material

HPLC-DAD chromatograms of phenolic compounds in *Myrtus communis L.* sample's extracts at (**A**) 280nm, (**B**) 360nm, and (**C**) 520nm: (**I**) Leaves, (**II**) Seeds, (**III**) fruits (Peel+Pulp).

(1) Gallic acid; (2) Myricetin 3-O-galactoside; (3) Vitexin; (4) Myricetin 3-O-rhamnoside; (5) Quercetin 3-O-galactoside; (6) Quercetin 3-O-glucoside, (7) Quercetin 3-O-rhamnoside; (8) Ellagic acid; (9) Cyanidin 3-O-glucoside; (10) Petunidin 3-O-glucoside; (11) Peonidin 3-O-glucoside; (12) Malvidin 3-O-glucoside; ($) Hydrolisable tannins

I

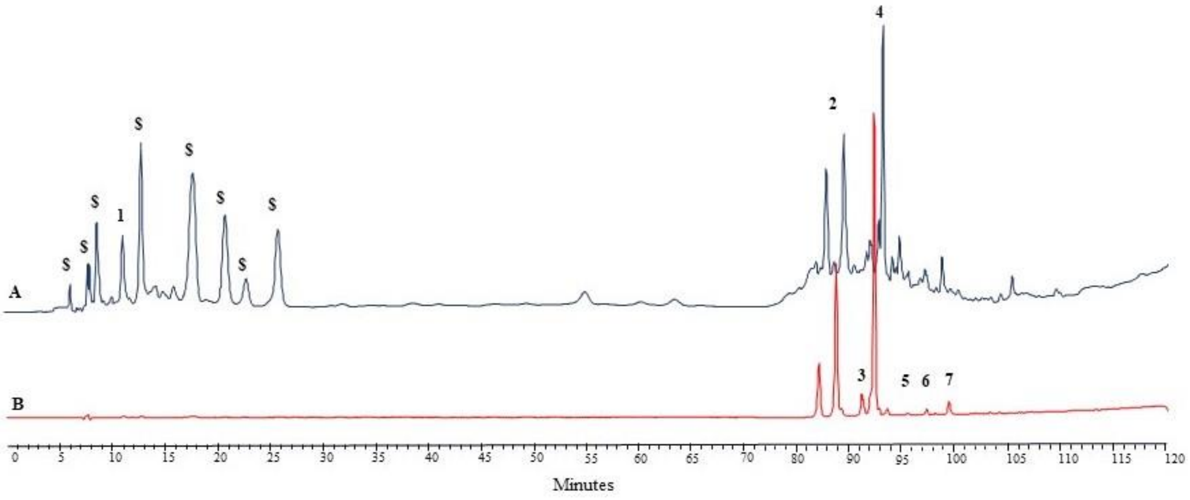

II

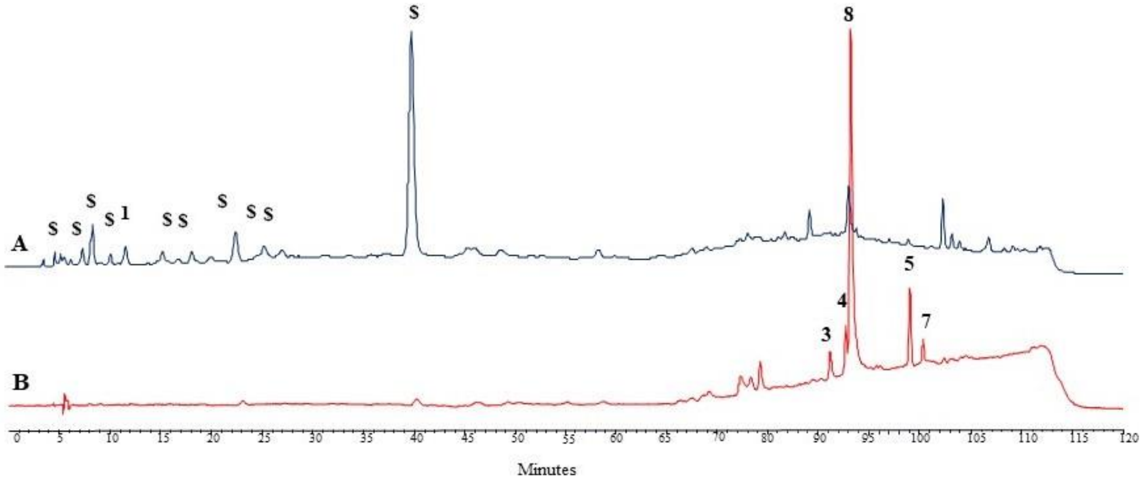

III

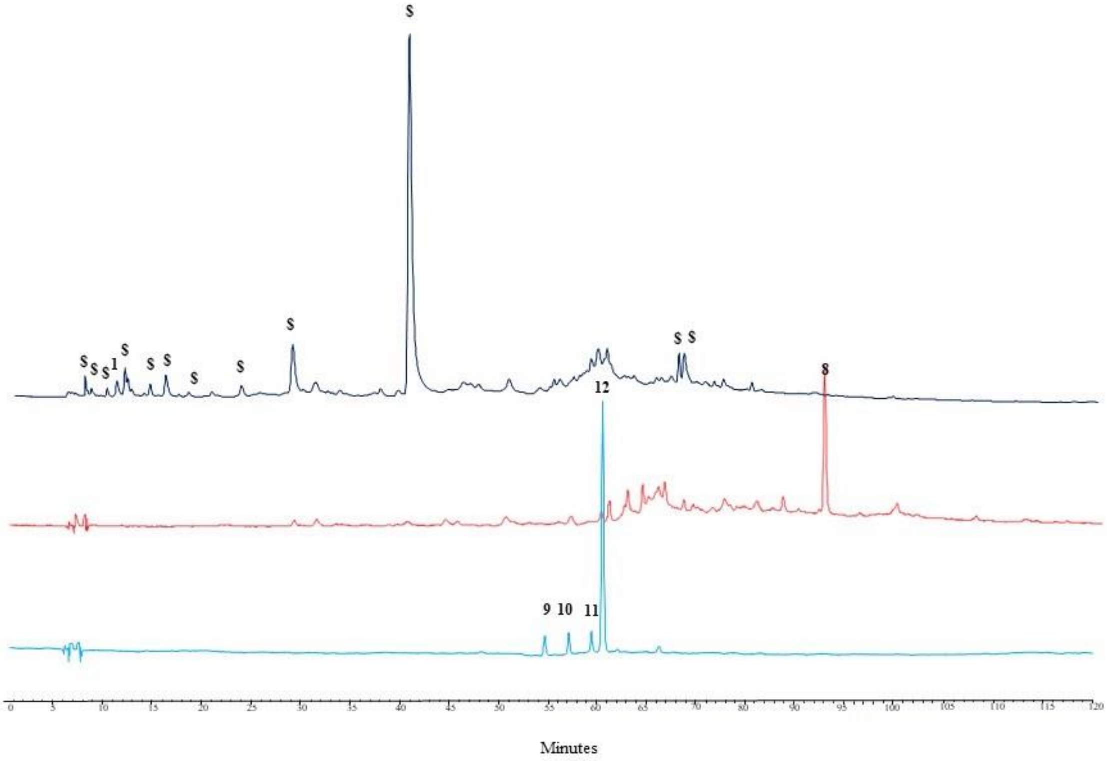

$
$ $ $ 1 $
$ $
$
$
$
$ $
8
12
9 10 11
0 5 10 15 20 25 30 35 40 45 50 55 60 65 70 75 80 85 90 95 100 105 110 115 120
Minutes
